# PERSON-CENTERED CARE PRACTICES PREDICT STAFF BURNOUT AND ENGAGEMENT IN LONG-TERM CARE STAFF

**DOI:** 10.1093/geroni/igae098.0068

**Published:** 2024-12-31

**Authors:** Sophia Geisser, Kate Smith, A Lynn Snow, Amber Collins, Kimberly Curyto, Christine Hartmann, Cameron Camp, Michelle Hilgeman

**Affiliations:** University of Alabama, Tuscaloosa, Alabama, United States; University of Alabama, Tuscaloosa, Alabama, United States; University of Alabama, Tuscaloosa, Alabama, United States; Tuscaloosa VA Medical Center, Tuscaloosa, Alabama, United States; Western New York VA Healthcare System, Batavia, New York, United States; VA Bedford Healthcare System & University of Massachusetts Lowell, Bedford, Massachusetts, United States; Center for Applied Research in Dementia, Solon, Ohio, United States; Tuscaloosa VA Medical Center, Tuscaloosa, Alabama, United States

## Abstract

Long-term care (LTC) staff experience above-average rates of burnout, which have personal and professional consequences. Little is known about the effects on staff of implementing person-centered care practices to improve LTC residents’ quality of life. We examined the relationship between staff perceptions of person-centered care and staff burnout and engagement in Veterans Affairs Community Living Centers (CLCs). We used baseline staff survey data (N=205) from a randomized Montessori implementation-effectiveness clinical trial in eight CLCs across five states. Data were collected anonymously online across all shifts (day/evening/night) from RNs (37.6%), nursing assistants (31.2%), and a variety of staff job types (31.3%). GLM models were used to examine the association between Person-Centered Practices in Assisted Living subscales (PC-PAL; Workplace Practices, Social Connectedness, Individualized Care and Services) and burnout and engagement measures from existing VA administrative data. Omnibus tests significantly predicted indicators of employee burnout and engagement (p=.05-p<.001). Individualized Care and Services predicted all three burnout indicators: 1) feeling burned out (F=4.289, p=.04); 2), considering leaving the job this year (F=5.477, p=.02), and 3) the job is emotionally hardening (F=4.026, p=.046); and one of the three engagement indicators (F=4.793, p=.03). Workplace Practices predicted all three measures of positive engagement: 1) seeing job as more than a paycheck (F=5.167, p=.02); job-related accomplishments (F=5.772, p=.017); and “doing more than is actually required” (F=8.888, p=.003). Social Connectedness was not a predictor in any model. Results demonstrated positive outcomes of person-centered care practices include impacts on LTC staff burnout and engagement.

